# Anatomic Areas of Lipoframing in Breast Surgery

**DOI:** 10.7759/cureus.57216

**Published:** 2024-03-29

**Authors:** Gustavo Jimenez Muñoz Ledo, Hector Ortiz, Alba Mayra Padilla

**Affiliations:** 1 Plastic and Reconstructive Surgery, Phi Aesthetics, Leon, MEX; 2 Surgery, ABC Hospital, Santa Fe Campus, Mexico City, MEX

**Keywords:** breast frame, liposuction, fat, breast surgery, breast lipoframing

## Abstract

Breast lipoframing is a concept in breast surgery that encompasses the often-overlooked aspects of the mammary glands, such as the surrounding structures and adipose tissue within the thorax. By acknowledging the interplay between these components and recognizing the need for their simultaneous treatment, breast lipoframing aims to optimize surgical aesthetic outcomes. This article proposes a comprehensive definition of the surrounding mammary fat tissue by delineating the specific areas of the breast involved in the lipoframing technique. It presents a retrospective analysis of 554 female patients, revealing only one case of seroma and three cases of hematoma. Furthermore, we explore the application of liposuction as a means to effectively treat these areas and achieve superior results.

## Introduction

The ideal breast shape and contour of it vary widely worldwide depending on geographic areas, and socio-cultural and religious issues, and nowadays is related to fashion trends and some influencer personalities such as actresses or models defined the beauty current parameters [1,2]. The aesthetic appeal of a woman's breasts can significantly contribute to her self-perception and confidence, symbolizing aspects such as femininity, fertility, and sexuality.

For women, the pre-axillary fat located beneath the deltopectoral fold and the subaxillary fat are some of their main concerns, even when the size and shape of their breasts look nice [3,4]. That is why we want to introduce the term “breast lipoframing” to consider and try to improve all the areas around the breast as an important part of the same mammary surgery. 

The breast frame is everything around the breast that will directly impact its aesthetic appearance, as fat tissue, the most common amounts are pre-axillary fat, lateral or sub-axillary fat, and inferior fat [5,6].

From a medical point of view, even when there have been great efforts to try to standardize the ideal breast proportions, volume, and shape, the aesthetic results depend on particular anatomical characteristics, patient desires, and of course, the artistic sense of each plastic surgeon [2,4,7]. Most of the publications focus on the position and volume of the gland, but just a few consider the importance of the chest that holds the breast and the fat structures that circumscribe them [4,5]. Safe liposuction, a technique performed by Simeon Wall, should be considered to minimize vascular damage, contour deformities and have a better aesthetic outcome [8].

The breasts are not a thorax-fixed structure, they have movement, and change their shape according to the particular characteristics of the thorax, most women have natural asymmetries that make their breasts and thorax unique. That is why an aesthetic result depends on some other factors, such as thorax cage configuration, and the peculiarities of frame fat deposits around the breast [9,10].

## Materials and methods

A retrospective study was conducted from 2014 to 2019 in our private practice to analyze the aesthetic results. The inclusion criteria were any female patient who underwent breast surgery with complementary lipoframing and liposuction (some lipofilling) during the same procedure. They had no history of liposuction or lipofilling in these areas, chest deformities, or syndromes.

A total of 554 female patients were included, with an age range of 18 to 62 years (mean age of 32 years), all operated on by the same surgeon. Smokers were not included; if they were, patients were asked to quit smoking at least four weeks before and after surgery. In this study, only 12 patients (2.1%) had a history of smoking.

Patients underwent various breast surgeries, including breast reduction, mastopexies, mastopexies with augmentation, breast augmentation with implants, and breast reconstructions (Table 1).

**Table 1:** Breast surgeries performed

Procedure	Total
Breast reductions	32 (5.7%)
Mastopexies	63 (11.3%)
Mastopexies- aug	102 (18.4%)
Breast augmentation with implants	228 (41.1%)
Breast reconstructions	129 (23.2%)
Total	554 (100%)

The procedures were accompanied by liposuction in the breast frame and lipoinfiltration in the mammary region, following a prior evaluation of each patient. The lipoframing technique (liposuction and lipofilling) was performed according to the different areas that we identified as sites with the greatest amount of fatty tissue surrounding the breast. These areas were treated separately with the following principles: deep plane liposuction, deep and superficial plane liposuction, the zone of possible lipofilling, and a 'don't touch' area. This approach was based on each patient's chest anatomy, including rib cage and thorax size, fat tissue distribution, mammary gland characteristics, and skin quality [9].

In our anatomical approach, we characterize specific areas related to fatty deposits, the lipofilling zone, and the no-touch area, and detail the corresponding liposuction planes applied in each region. The delineation of these areas is guided by anatomical references as follows.

The preaxillary fat region is circumscribed by the anterior axillary line, deltopectoral groove, and the upper border of the upper mammary pole. Deep and superficial liposuction techniques are employed in this circular region.

Moving to the lateral or sub-axillary fat, we define a rectangle with the anterior and posterior axillary lines, the upper boundary being the base of the axillary hollow, and the lower boundary extending to the seventh intercostal space. Deep liposuction is carried out in this rectangular region.

The inferior mammary fat area is outlined as a rectangle with the inframammary fold as the superior limit, the anterior axillary line as the lateral limit, 1.5 centimeters lateral to the midsternal line as the medial limit, and the seventh intercostal space as the lower limit. For this region, deep and superficial liposuction techniques are applied, with the superior limit adjusting in cases of reduction or mastopexy.

Concerning the lipofilling area, we advocate for its performance upon request throughout the entire breast parenchyma. Finally, the no-touch area is designated as 1.5 centimeters from each side of the sternal midline. This area is treated with a hands-off approach during the surgical procedure.

Marking

**Figure 1:**
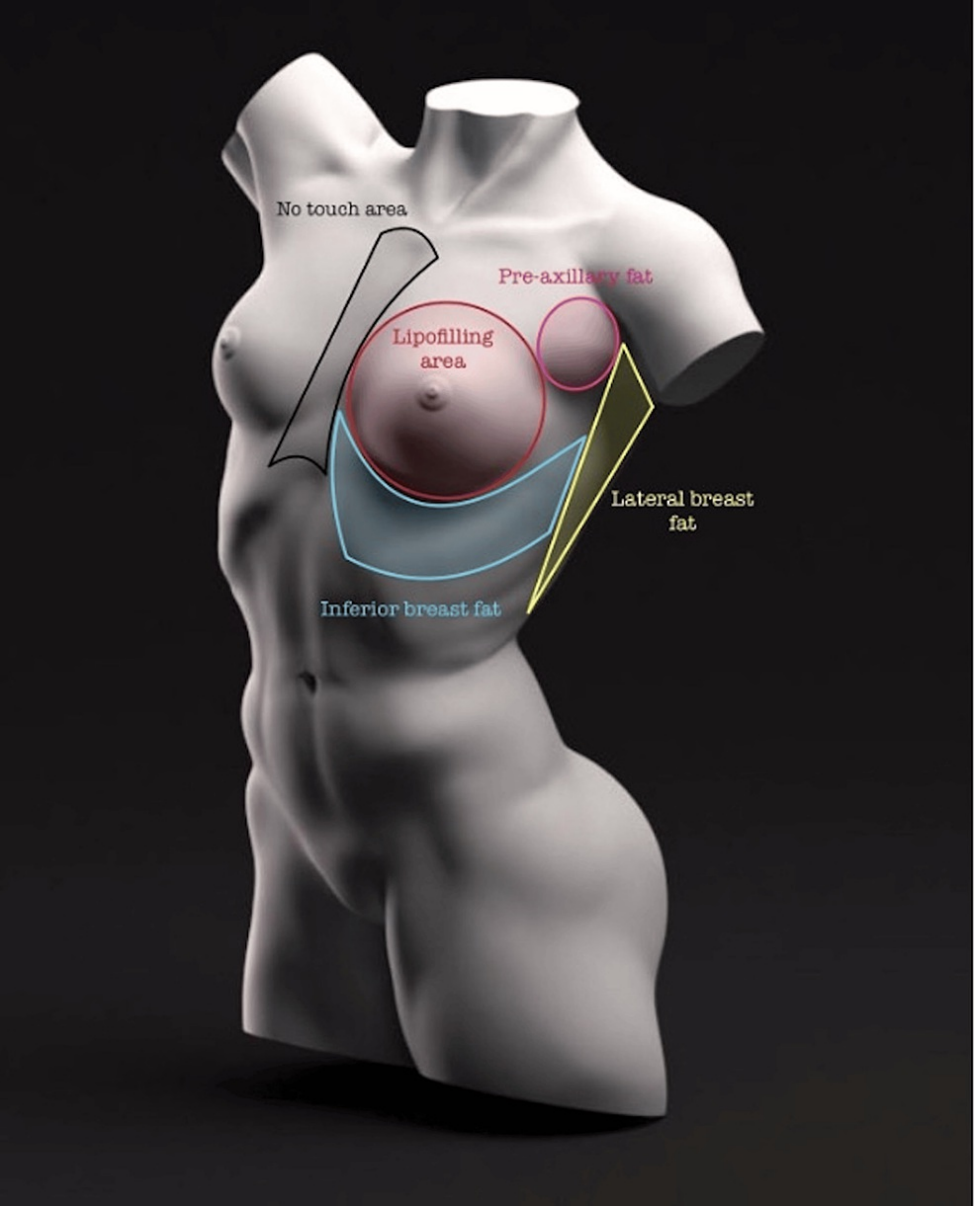
Proposition to define the anatomical areas surrounding the mammary region

**Figure 2:**
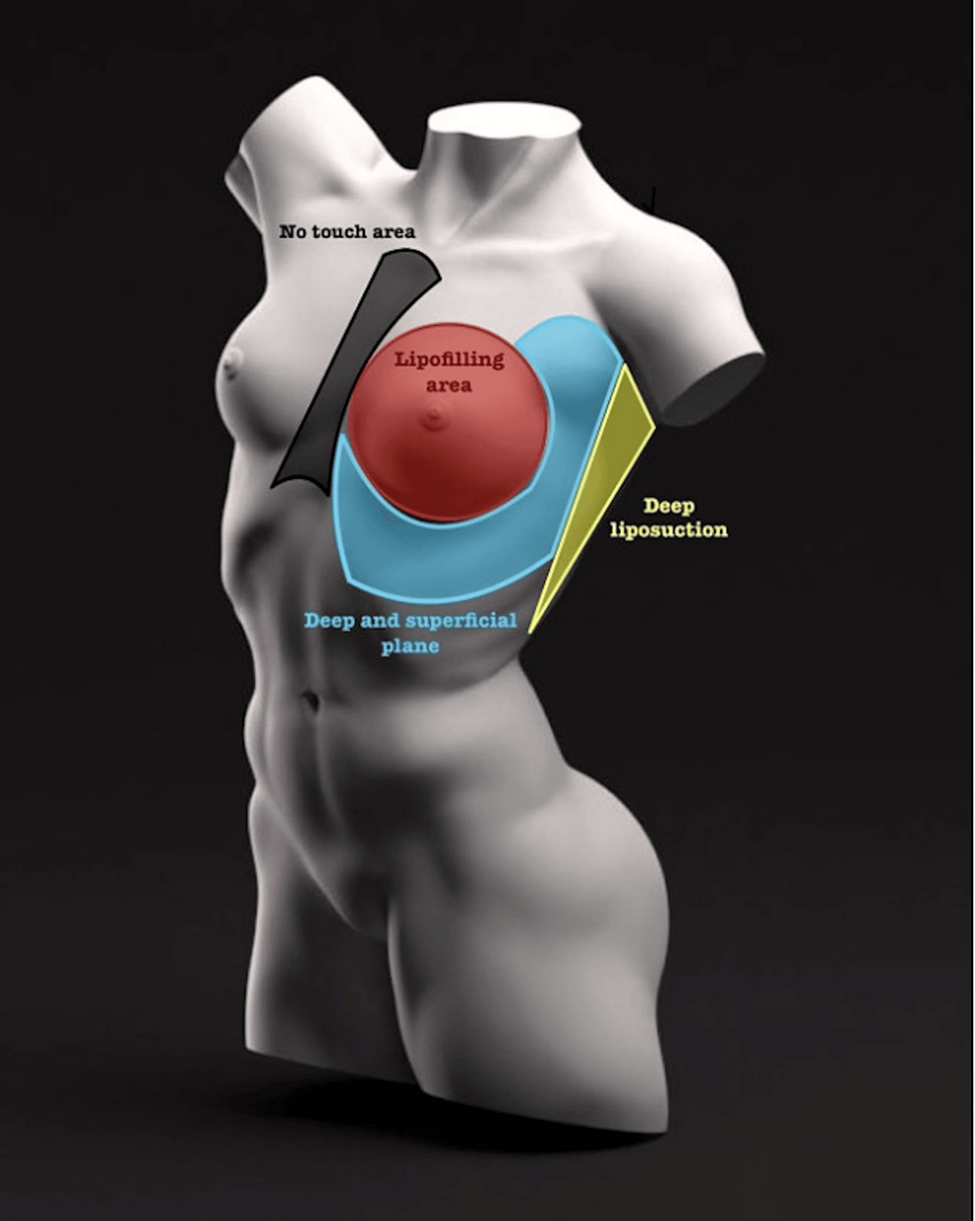
According to the previously delimited areas, we mention the liposuction plane in them

The lipoframing areas were marked previously with the patients standing, and with a dynamic exploration, this one is carried out in three steps, the first standing with the arms raised or at the nape of the neck, the second sitting with arms pressing on the hips and the third, sitting with a forward inclination, all three based on a more complete observation with an analysis of surrounding structures and asymmetry between both breasts. The approach was individualized in each case, and this is how we mark our anatomical areas surrounding the mammary region (Figures 1, 2).

Breast lipoframing technique

Once breast surgery is performed, the next step is the evaluation of the surrounding fatty tissue, how to treat it by liposuction, in which plane and possible lipofilling areas.

We take advantage of the wounds so we do not make extra incisions, trying not to communicate the surgical area with the cannula course, in order not to increase the risk of infections and failure or damage to breast surgery performed, especially in the use of alloplastics.

We used a tumescent solution with 1000cc of saline solution added with 1 ampule (1cc) epinephrine 1:1000, performing a super humid liposuction technique. The liposuction planes do not change whether traditional or power-assisted liposuction you prefer to do. 

We performed traditional liposuction in every case using a blunt Mercedes 3-4 mm cannula. First, we made the deep plane liposuction due to the greater amount of fatty tissue, the same that when reduced, shows in a more significant way the anatomy of the breast. Second, we made the superficial planes to delimit the breast print, in this step the deep plane should be liposuctioned in less quantity, seeking an adequate transition of the breast with the surrounding adipose tissue.

The amount of fat suction was different in each patient according to their fat deposits, being from 50 to 400cc (25-50 cc pre-axillary, 50-150 cc lateral breast fat, 25-150 cc inferior). The fat was prepared at first by washing it with an antiseptic solution base on hypochlorous acid, sodium hypochlorite, and sodium phosphate, then by decanting it, an antibiotic (aminoglycoside) was added, and injecting it into the mammary parenchyma and subcutaneous tissue.

We do not use drainage independently on the type of breast surgery and the lipofilling. The wounds were covered with microporous adhesive tape. The use of bandages was individualized to each breast surgery.

The patients were discharged the same day in the cases of only breast surgery, cases in combination with body contouring, their hospitalization was for one day.

Dermato-funcional management

**Figure 3:**
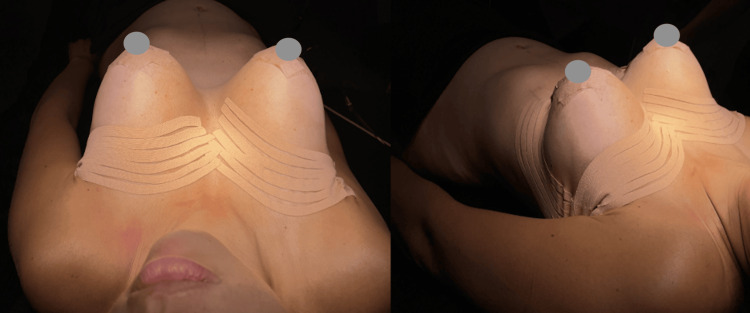
Lymphatic breast taping

Lymphatic taping technique is used, it has been performed by Chi et al. during the transoperative period to reduce postoperative ecchymosis, pain and edema. It is retired seven days after surgery (Figure 3) [11].

Postoperative management

After surgery, we used lymphatic taping and compression garments together, divided into two phases based on their compression grade as follows: phase 1: mild compression, 24 hours first two weeks; phase 2: moderate compression, 24 hours, third and fourth week. Then the same garment 12 hours a day two weeks more. After completing six weeks of it the use of garment stopped and hypopressive exercises started. 

In terms of nutritional counseling after a plastic procedure diet plays an important role, we advise a low carbohydrate diet to decrease proinflammatory factors, metabolic trauma response and have better wound healing. Moreover studies are needed in terms of macronutrients diet assessment after aesthetics surgeries.

Then the patients were under lymphatic drainage and ultrasound starting the fourth day after surgery with 10 sessions in total every 48-72 hours.

## Results

It was a total of 554 female patients who underwent lipoframing after their breast surgery, based on the anatomical zones that we describe and how to treat them. We had a total of 214 (38.6%) lipofilling cases that we infiltrated from 50cc to 400cc in order of breast symmetrization. Complications were one case of a hematoma, three cases of seroma, and in some cases the presence of ecchymosis was found without clinical repercussions. We did not have bleeding, dehiscence, infections, fat necrosis, rupture of the implant, ribs, and lung issues, etc. 

**Figure 4:**
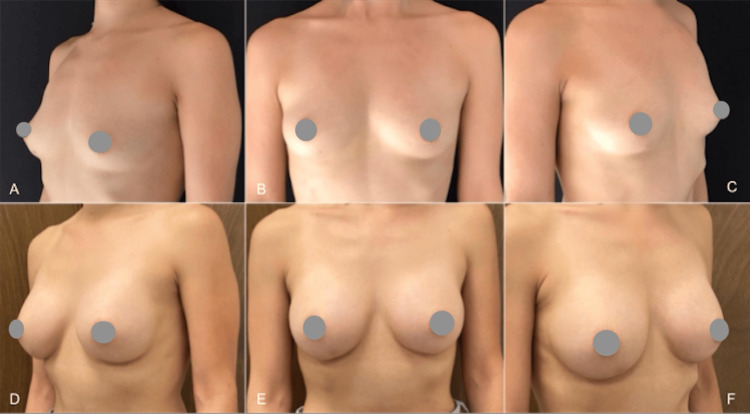
A case of mammoplasty with 310cc implants and pre-axillary liposuction. Photos before surgery (A-C) and 14 months after (D-F)

In the first case, a 24-year-old woman underwent mammoplasty with 310cc breast implants, accompanied by pre-axillary super-wet liposuction (65cc on the left and 78cc on the right). Preoperative and postoperative photos, taken 14 months apart, are presented in Figure 4.

**Figure 5:**
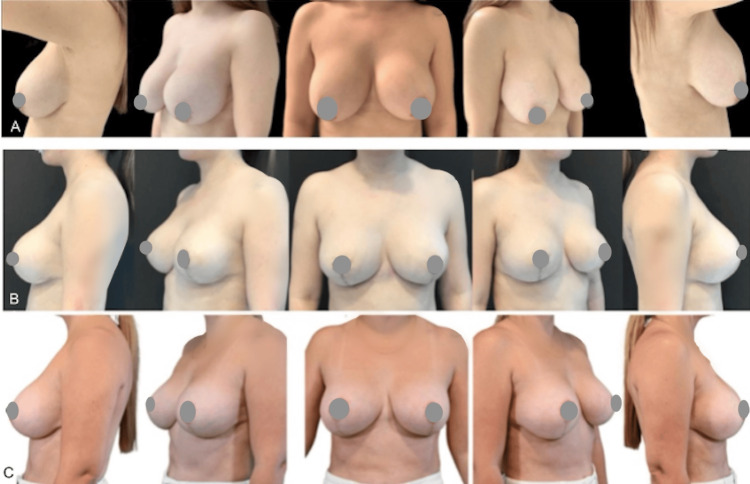
A case involving a patient with previous mammoplasty with implants and waterwall deformity underwent mastopexy with implant replacement, along with liposuction of the arms, lateral breast, and pre-axillary region. Photos before surgery (A), 25 days after surgery (B), 13 months after surgery (C)

The second case involves a 42-year-old woman with a waterfall deformity who underwent mastopexy with a change in breast implants to a subfascial plane. Additionally, super-wet liposuction was performed on the arms (220cc on the left, 225 cc on the right), lateral breast (110cc on the left, 120cc on the right), and pre-axillary region (45cc on the left, 55cc on the right). Photos taken pre-surgery photos (A), images taken 25 days post-surgery (B), and those captured 13 months after the procedure (C) (Figure 5).

**Figure 6:**
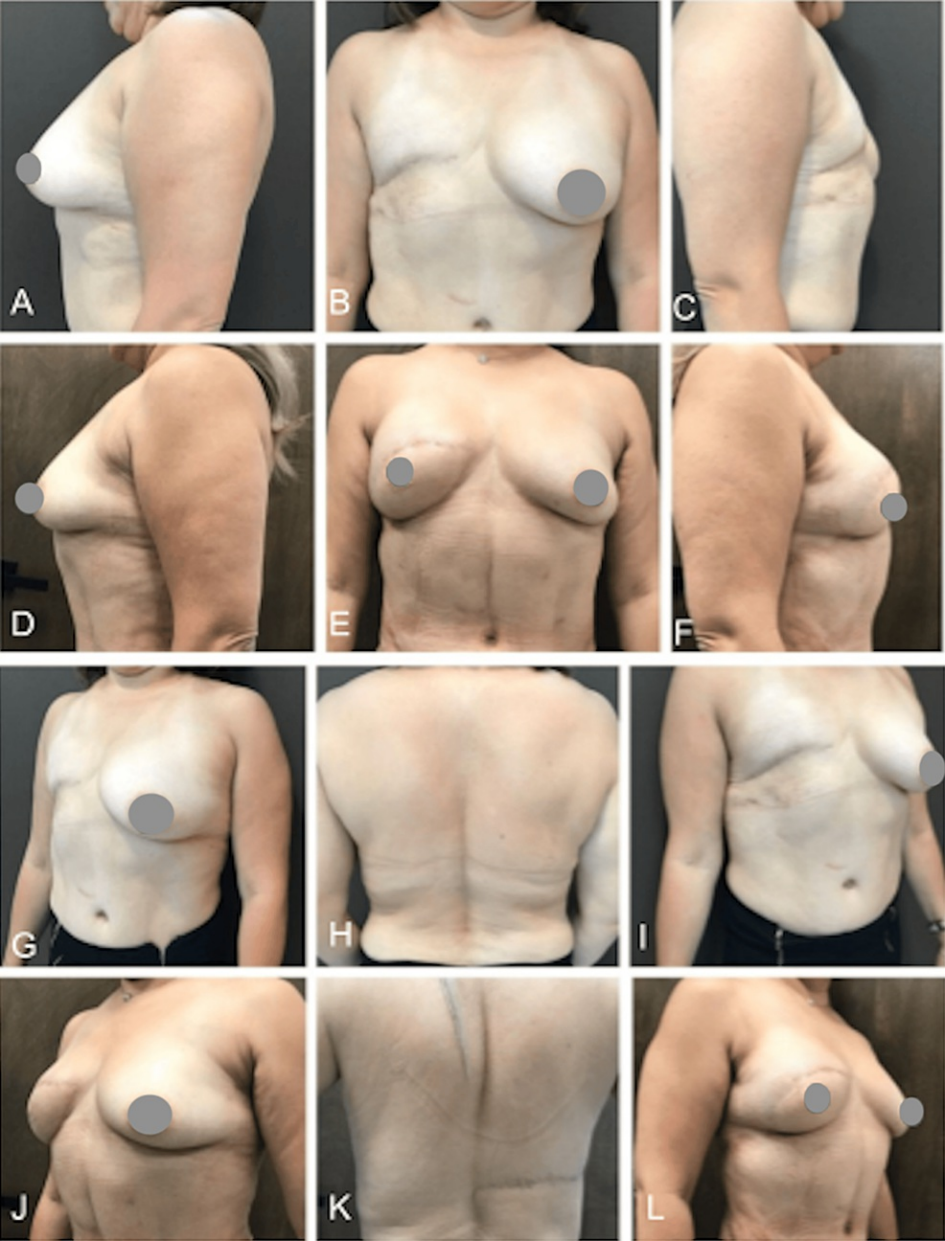
A case involving a history of breast cancer with reconstruction in two phases: the first phase utilized a dorsal-epigastric right flap, and the second phase involved pre-axillary, left thoracic, and back liposuction followed by lipofilling in the superior quadrant of the breast and pre-axillary region. Photos pre surgery (A-C and G-I) and after 14 months (D-F and J-L)

The third case involves a 53-year-old woman with a history of breast cancer who underwent breast reconstruction in two phases. The first phase included a dorsal-epigastric right flap, and in the second phase, performed 10 months later, super-wet liposuction was carried out on the left pre-axillary (140cc), left thoracic (220cc), and back (400cc) regions. Lipofilling via a microdroplet and vibration technique was applied, with 400cc in the breast's superior quadrant and 300cc in the pre-axillary downfall. Preoperative and 14-month postoperative photos are depicted in Figure 6. 

**Figure 7:**
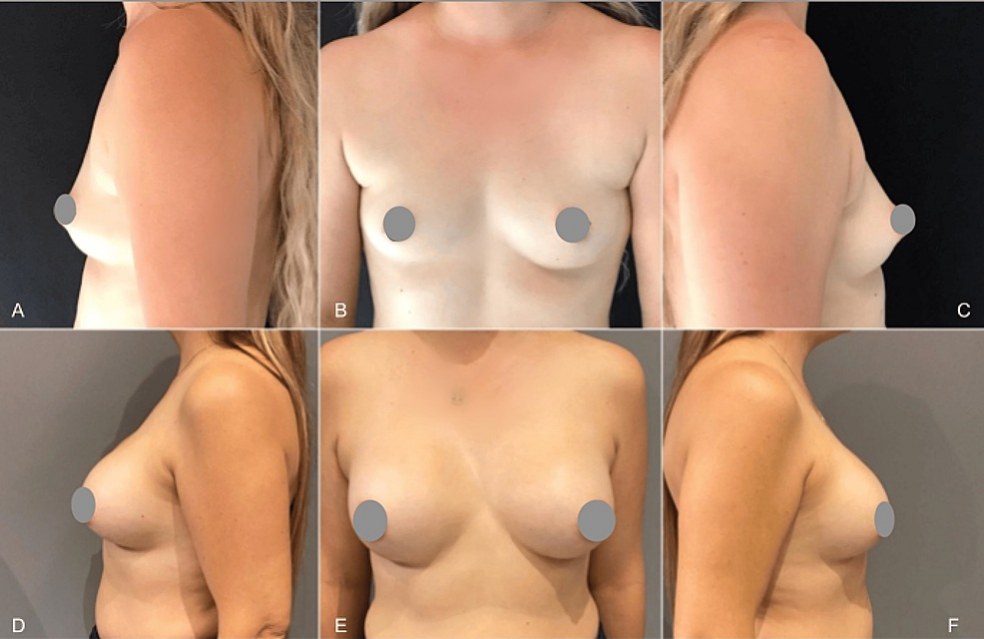
A case with breast asymmetry and tuberous deformity underwent mammoplasty with implants and pre-axillary liposuction. Photos pre surgery (A-C) and after 14 months (D-F)

In the subsequent case, a 25-year-old woman with breast asymmetry and tuberous deformity grade I underwent mammoplasty with 340cc implants. Super-wet pre-axillary liposuction was performed, with 75cc on the left and 65cc on the right. Pre-surgery and 14-month postoperative photos are displayed in Figure 7. 

**Figure 8:**
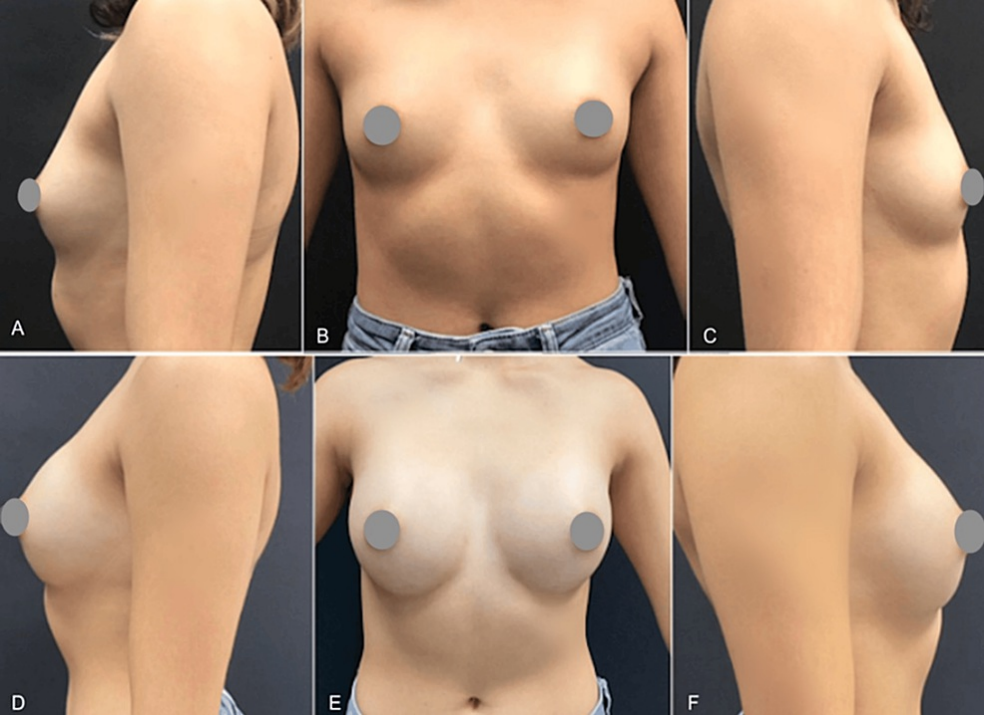
A case of mammoplasty with 200 cc implants and pre-axillary liposuction. Photos pre surgery (A-C) and after six months (D-F)

The final case involves a 26-year-old woman who underwent mammoplasty with 200cc round smooth implants, along with pre-axillary liposuction (60cc on the left and 50cc on the right). Preoperative and six-month postoperative photos are presented in Figure 8.

## Discussion

The integral approach to a patient requesting breast surgery should include the evaluation of the breast frame, encompassing considerations such as breast footprint, ptosis, and skin envelope. Surgeons may delve into various surgical techniques and principles aimed at achieving optimal breast aesthetics, potentially addressing the role of liposuction and its impact on surrounding fat tissue. Hall-Findlay and Evans likely contribute valuable insights into the nuanced artistry of breast surgery, emphasizing the need for a tailored approach that takes into account individual variations in breast anatomy and the surrounding adipose tissue [12].

The presented research paper outlines the outcomes of lipoframing procedures in 554 female patients following breast surgery. The study, which specifically focuses on anatomical zones and their treatment, reports a significant percentage of lipofilling cases, totaling 38.6% of the patient cohort. The infiltration volumes ranging from 50cc to 400cc for breast symmetrization demonstrate a tailored approach to achieving aesthetic results. Notably, the study highlights a low incidence of complications, with only one case of hematoma and three cases of seroma. Some cases exhibited ecchymosis without clinical repercussions, while more severe complications such as bleeding, dehiscence, infections, fat necrosis, and issues related to implant rupture, ribs, and lungs were notably absent.

By incorporating breast lipoframing, we can also consider breast footprint, areola, conus, and skin envelope described by Martinovic and Blancet with the mnemonic BFACE but also discussed by Blondeel et al. [3,13]. Swanson's 2015 study underscores the importance of understanding patient preferences in shaping the outcomes of breast surgery, emphasizing the significance of convexity and upper pole fullness. This understanding may influence decisions related to liposuction and lipofilling in the surrounding breast fat tissue [2].

Ideas from Hoyos, Malluci, and Simeon Wall Jr. lead us to pay attention to every detail, considering the steps and recommendations in different types of breast surgery. These diverse insights converge into one constant: making a comprehensive morphometric analysis of the breast framework and its surrounding fat tissue for optimal surgical outcomes [8,14]. In 2017, Mistry et al. and MacLennan and Hall-Findlay highlighted the nuanced understanding of breast surgery re-reduction, enabling surgeons to gain valuable insights into refining their approaches to liposuction and addressing the surrounding breast fat tissue during secondary breast surgeries [5,15].

That is why, in every single procedure, we delineate the fat tissue surrounding the breast and seize the opportunity to reduce it with the aim of enhancing the breast contour. This process takes into account the basic principles of liposuction, considering planes, the amount, and density of fatty tissue. We perform both deep and superficial liposuction in specific areas, intending to create negative spaces where needed, such as the pre-axillary and inferior fat regions, as well as the midline area. In the sub-axillary fat region, our goal is reduction while preserving some fat tissue to protect the nerve pathway. Recognizing the inherent asymmetry between breasts, the decision to utilize lipofilling is made in an effort to achieve the greatest possible symmetry.

Limitations of study

The paper presents a comprehensive approach to breast surgery, its applicability to diverse patient populations, the absence of standardized outcome measures, and the limited long-term follow-up data should be considered as important limitations that may impact the generalizability and robustness of the study's conclusions.

## Conclusions

The breast frame should be analyzed in preoperative consultation; in those cases that distort the breast aesthetics, we should plan to make some liposuction and contouring on them to achieve a superior aesthetic result that gives harmony to the thorax as a unit. 

Identification and limitation of the fat areas that surround the breast are of great importance, as the shape and size of the thorax are so that when manipulating them, we can highlight the beauty of the breast by creating the ideal frame to give the best result.

A comparative study is needed, under the same breast surgical procedure and similar anatomical characteristics in patients who performed breast lipoframing and those who did not, evaluating the result on a satisfaction scale for the patients and the surgeon doing it double-blind. It will be necessary to continue the follow-up of these patients for more than five years to evaluate the evolution.
